# Increased temperature disrupts chemical communication in some species but not others: The importance of local adaptation and distribution

**DOI:** 10.1002/ece3.3646

**Published:** 2017-12-15

**Authors:** Maider Iglesias‐Carrasco, Megan L. Head, José Martín, Carlos Cabido

**Affiliations:** ^1^ Department of Evolutionary Ecology National Museum of Natural Sciences Spanish National Research Council (CSIC) Madrid Spain; ^2^ Department of Herpetology Aranzadi Science Society Donostia‐San Sebastián Spain; ^3^ Division of Evolution, Ecology and Genetics Research School of Biology Australian National University Canberra ACT Australia

**Keywords:** altitude, chemical signals, communication, lizard, temperature, tongue flick

## Abstract

Environmental conditions experienced by a species during its evolutionary history may shape the signals it uses for communication. Consequently, rapid environmental changes may lead to less effective signals, which interfere with communication between individuals, altering life history traits such as predator detection and mate searching. Increased temperature can reduce the efficacy of scent marks released by male lizards, but the extent to which this negative effect is related to specific biological traits and evolutionary histories across species and populations have not been explored. We experimentally tested how increased temperature affects the efficacy of chemical signals of high‐ and low‐altitude populations of three lizard species that differ in their ecological requirements and altitudinal distributions. We tested the behavioral chemosensory responses of males from each species and population to male scent marks that had been incubated at one of two temperatures (cold 16°C or hot 20°C). In high‐altitude populations of a mountain species (*Iberolacerta monticola*), the efficacy of chemical signals (i.e., latency time and number of tongue flicks) was lower after scent marks had been exposed to a hot temperature. The temperature that scent marks were incubated at did not affect the efficacy of chemical signals in a ubiquitous species (*Podarcis muralis*) or another mountain species (*I. bonalli*). Our results suggest that specific ecological traits arising through local adaptation to restricted distributions may be important in determining species vulnerability to climatic change.

## 1 INTRODUCTION

Environmental conditions that species experience over their evolutionary history are important for shaping the evolution of their signals (i.e., traits that, due to their adaptive selection, provide information that might change the behavior of receiver individuals, Zahavi, 1991), and may lead signal transmission and detection to become locally adapted (Endler, 1992). Local adaptation, including genetic variation and/or phenotypic plasticity, has been demonstrated for visual signals. For example, male *Anolis cristatellus* lizards inhabiting habitats with different light intensity and spectral quality have different reflectance and transmittance dewlap design to increase signal detectability in each habitat (Leal & Fleishman, 2004). Likewise, acoustic signals such as bird song are often adapted to the habitat in which a species has evolved, and song transmission has been shown to be less efficient in novel habitats (Nemeth, Winkler, & Dabelsteen, 2001).

Rapid changes in the environment (e.g., increased background noise) could alter the expression, transmission, or even the detection of signals. Such environmental changes could interfere with, or prevent species from being able to perform daily tasks that are essential for their survival and reproduction (Fabian, Albright, Gerlach, Fisher, & Rosenthal, 2007; Iglesias‐Carrasco, Head, Jennions, Martín, & Cabido, 2017; Wolf & Moore, 2002). Human‐induced disturbances, for instance, can disrupt several sensory modalities, such as visual, acoustic, or chemical, and thus may affect important processes from sexual selection to predator–prey interactions. Examples of how environmental change can impair communication can be found both in aquatic and in terrestrial organisms. For example, increased turbidity due to the presence of algae alters the visual environment in which male three‐spine sticklebacks *Gasterosteus maculatus* seek mates. Reduced visibility of their red nuptial coloration decreases male attractiveness to potential mates and triggers increased investment in this costly visual signal (Engström‐Öst & Candolin, 2006). Similarly, anthropic noise can alter the acoustic background against which birds sing to attract mates, call to warn of predators, and beg to elicit feeding from caring parents. Such noise can reduce signal detection through “sound masking” (e.g., Engström‐Öst & Candolin, 2006) and limit the distance at which individuals can communicate (Nemeth & Brumm, 2010).

Individuals of different species and populations can differ in their response to environmental change (Williams, Shoo, Isaac, Hoffmann, & Langham, 2008). Some human‐created novel habitats, for example, have conditions that are similar to the original habitats. Therefore, individuals from some species and/or populations exhibit preadaptations to these environmental modifications, and so are better able to invade the newly created habitats (Blackburn, Lockwood, & Cassey, 2009). For example, generalist bird species, with broad habitat or dietary requirements, are more likely to successfully establish in new environments than are specialist species, because generalists are more likely to find the necessary conditions and resources in the novel habitat (Cassey, Blackburn, Sol, Duncan, & Lockwood, 2004). Further, some biological characteristics may predispose individuals from different populations to be more vulnerable to environmental change and thus might predict the likelihood that individuals respond to novel conditions (Foden et al., 2013). For example, limited dispersal ability (Hamer & McDonnell, 2008), restricted geographic distribution (Foden et al., 2013), and narrow physiological tolerances (Buckley, 2010) can all increase the vulnerability of species and populations to environmental change, so they become more prone to extinction than others.

Chemical communication is an important sensory modality that is used by many terrestrial and aquatic species to successfully perform a range of activities that enhance their ability to survive and reproduce (Cross, Blumstein, & Rosell, 2013; Lemasson, Mikus, Blois‐Heulin, & Lodé, 2013; Wyatt, 2014). For example, many organisms use olfaction for interspecific communication, which is essential for recognizing and discriminating between group and nongroup members (Billingham, Chapple, Sunnucks, & Wong, 2009) or for identifying potential competitors (Carazo, Font, & Desfilis, 2008). Further, both aquatic and terrestrial species rely on chemical signals to identify potential mates (e.g., Wyatt, 2014). In aquatic habitats, the disruption of this sensory modality driven by the alteration of the chemical environment has been widely explored (reviewed in Billingham et al., 2009). For example, in the crayfish *Cambarus bartonii*, a sublethal exposure to copper impaired the odor‐mediated localization of prey, increasing the latency times (LTs) to find food items (Sherba, Dunham, & Harvey, 2000). Similarly, female palmate newts, *Lissotriton helveticus*, failed to associate with mates of higher quality after long‐term exposure to the secondary compounds of an exotic tree (Iglesias‐Carrasco, Head, Jennions, & Cabido, 2017). In terrestrial organisms, studies of environmentally induced disruption of chemical communication are scarce in several taxonomic groups with the exception of insects (Komeza, Fouillet, Boulétreau, & Delpuech, 2001; Wei & Du, 2004). However, we have little understanding of how environmental change disrupts chemical communication in terrestrial vertebrates (but see Iglesias‐Carrasco, Head, Jennions, & Cabido, 2017; Komeza et al., 2001).

Reptiles are an interesting target group to study disruption of chemical communication. In many lizards, chemical signals are essential for both female mate choice (Olsson et al., 2003) and for intrasexual relationships between males (Carazo, Font, & Desfilis, 2007). In the case of lacertid lizards, males produce chemical signals that are deposited on the substrate as scent marks (Martín & López, 2014). These scent marks are exploited by both females and males, to get information about their conspecifics’ general condition (Martín & López, 2015; Mason & Parker, 2010). These chemical secretions seem to be highly tuned to the temperature and humidity of the environment in which they evolved (Martín & López, 2013; Martín, Ortega, & López, 2015), so alteration of these environmental characteristics might strongly influence the efficacy of these chemical secretions.

In terrestrial environments, the temperature in which chemical signals evolved can determine the composition and relative amount of each compound in a signal to maximize the transmission, durability, and persistence of the signal (Alberts, 1992; Apps, Weldon, & Kramer, 2015). Thus, interspecific differences in chemical signal composition might reflect selection for the efficacy of signals in different environmental temperature conditions (Escobar, Labra, Niemeyer, & Escobar, 2003). For example, the composition of chemical signals of the Iberian wall lizard, *Podarcis hispanica*, differed between two populations inhabiting environments with different climatic conditions (Martín et al., 2015). Lizards from the lower altitude population inhabiting hotter climatic conditions had secretions with higher proportions of cholesterol and fatty acids, but lower proportions of alcohols than Lizards from the lower altitude population inhabiting hotter climatic conditions had secretions with higher proportions of cholesterol and fatty acids, but lower proportions of alcohols than lizards from the higher altitude that are exposed to colder temperatures. could lead to a higher volatilization and degradation of chemical compounds, thus reducing the efficacy of chemical signals in warmer habitats (Martín et al., 2015). Whether such results are dependent on species ecology or evolutionary history, however, has not been explored.

Here, we experimentally explored whether the detectability of femoral gland secretions of male lizards was affected by the exposure of these secretions (i.e., scent marks) to different temperatures. We explored this question in two populations inhabiting different altitudes of each of three lizard species with marked ecological differences; we chose two mountain species (*Iberolacerta monticola* and *Iberolcarta bonnalli*) with restricted geographic distributions and one ubiquitous species (*Podarcis muralis*) with a wide geographic distribution. Lizard chemical signals may, to some extent, reflect a selection process or phenotypic plasticity to maximize the efficacy of signals in different climates and/or habitats (Alberts, 1992; Martín, Javier Zamora‐Camacho, Reguera, López, & Moreno‐Rueda, 2017) If this was the case, we expected differences between populations and species in the chemical composition of femoral gland secretions (Martín et al., 2015) that may lead scent marks of some populations to be more sensitive to differences in temperature than others. To test for differences between populations in the efficacy of chemical signals after the exposure of these scent marks to different temperature treatments, we compared populations of each species inhabiting different altitudes. We examined the chemosensory responses (i.e., tongue‐flicking behavior) of males to scent marks that had been exposed for 4 hr to cold (16°C) or hot (20°C) temperatures. We predicted that: (1) the efficacy of the chemical signals will be lower after the exposure to high temperatures compared to the lower temperature, but that (2) the negative effects of the increase in temperature will be more detrimental for the mountain species than for the ubiquitous species, and (3) within each species, the reduction in signal efficacy will be sharper for the populations inhabiting higher altitudes.

## 2 METHODS

### 2.1 Study species

We captured males of three lacertid lizard species to test for the effects of temperature on the durability and detectability of scent marks. We selected two mountain species of the genus *Iberolacerta* and a widespread species of the genus *Podarcis*. The three species were chosen because they are lacertid lizards with similar morphology, and habitat requirements that, in some places, cohabit in sympatry. However, they differ in several ecological traits, such as their altitudinal distribution, mating system, and their thermal tolerances. *Podarcis muralis* is widely distributed, inhabiting a range of habitats (including rocky seaside cliffs to mountain forests and urban areas) from sea level to 2,400 m altitude (Pleguezuelos & Villafranca, 1997), from the Iberian Peninsula to the Black sea coasts and North Turkey (Salvador, 2014). In some populations, males are territorial and copulate with females that enter their defended areas (Brown, Gist, & Taylor, 1995). While in other populations of this species, the overlap of home ranges suggests a hierarchical social system (Edsman, 1991). *Iberolacerta monticola* is endemic to the Iberian Peninsula, with a distribution reduced to the Cantabric Mountains and some more isolated sites in Galicia and North Portugal. This species is typically found in high mountain rocky habitats, surrounded by scrub and grassland. Although it is more common in altitudes ranging from 1,400 to 2,000 m, there are also small populations at the sea level. It is a threatened species (Pérez‐Mellado, Sá‐Sousa, Marquez, & Martínez‐Solano, 2009), mainly due to the destruction of mountain habitats. It is a polygynous species, where older males defend a territory which varies in size depending on the population density (Moreira, Almeida, Delgado, Salgueiro, & Crespo, 1998). *Iberolacerta bonalli* is limited to high mountain rocky outcrops in the Pyrenees Mountains and inhabits altitudes ranging from 1,700 to 3,000 m (Pottier, 2001). This species is threatened due to a reduction in habitat availability resulting from the destruction of mountain habitats and climate change. This species has limited territoriality, and agonistic fights between males are rare (Martínez‐Rica, 1977).

### 2.2 Collection and maintenance of lizards

We captured adult male lizards of each species in two populations located at different altitudes (low and high), which were selected based on the elevational distribution ranges of each species: *P. muralis* were collected at 600 m asl (Picos de Europa mountains, 43°26′ 19.24″N, 5°11′35.70″W) and 2,000 m asl (Pyrenees, 42°37′43.53″N, 0°01′31.43″E); *I. monticola* were collected in Picos de Europa mountains at 600 m asl (43°26′19.24″N, 5°11′35.70″W) and 1900 m asl (43°09′52.28″N, 4°51′35.42″W); and *I. bonnali* were collected in the Pyrenees mountains at 1,900 m asl (42°39′03.68″N, 0°00′52.89″E) and 2,400 m asl (42°39′57.18″N, 0°01′05.04″E). All lizards were captured by harmless noosing during their reproductive season (May–July) in 2013. In each population, we captured 20 to 24 males. Ideally, we would have liked to collect and test males from more than one population per altitude, however, the restricted geographic distribution, the difficulty in accessing some populations and the endangered status of the mountain species made it very difficult to capture more individuals.

Upon capture, lizards were transported to the laboratory where they were individually housed in indoor 36 × 42 cm PVC terraria containing rocks for refuge, a heat bulb and a UV‐lamp, situated at one end of each terrarium. This ensured an air temperature gradient (21–37°C) that allowed lizards to freely attain and select their preferred body temperatures. The photoperiod mimicked that of the surrounding region. We watered and fed animals mealworm larvae and crickets dusted with multivitamin powder ad libitum. Lizards were allowed 3 days to acclimate to their new surroundings prior to testing. All the animals were healthy, and they were returned to their capture sites at the end of the trials, 2 weeks after being captured.

### 2.3 Experimental design

**Figure 1:**
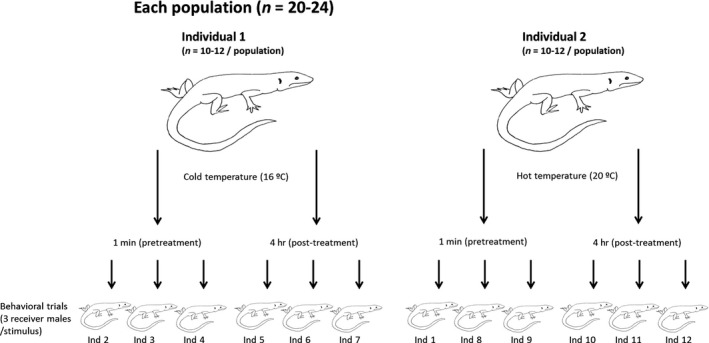
Experimental design. We used two swabs to collect femoral secretions from each male of each population. Thereafter, swabs from each male were incubated for a short (1 min) or a long (4 hr) period of time at either hot (20°C) or cold (16°C) temperature. Each of the swabs was presented to three corresponding conspecific males of the same population

To test whether the efficacy of chemical signals depends on the temperature to which these signals were exposed after being deposited as scent marks, and whether these effects differ depending on the altitude a lizard comes from or the species ecology, we tested the behavioral responses of males from each species and population to the scent of conspecific males from their own population. Scents were obtained by swabbing male femoral glands, and the swabs were then incubated at two different temperatures: cold (16°C) or hot (20°C), for either a short (1 min) or a long (4 hr) period (Figure 1). Little is known about the thermal biology of our study species in their natural habitats, as such our aim was not to try and relate our results to specific temperatures that each population might experience in the field, but rather to simply explore whether secretions of different species and populations are responded to differently when they have been incubated at different temperatures. The temperatures were chosen to be comparable with previous studies in similar species (Martín & López, 2013; Martín et al., 2015).

### 2.4 Secretion preparation

We extracted femoral secretions from all males that showed active femoral pores (20 to 24 males from each population of each species). The number of individuals used was restricted by how many lizards we were able to capture in the high‐altitude populations. To collect secretions, we pressed around the femoral pores and collected the waxy secretion directly with the cotton tip of a wooden applicator. To avoid the possibility that differences in the response of focal males was motivated by different amounts of secretion, we standardized the quantity of secretion collected by swabbing the secretion from three pores per male (1 × 2 mm of waxy secretion for each of the three pores, see Iglesias‐Carrasco, Head, Jennions, & Cabido, 2017). We collected two swabs per male, one from each hindlimb.

After the collection of secretions, the cotton swabs were placed in an incubator at either 16°C (cold treatment) or 20° (hot treatment). We used the same temperature ranges for the three species and all the populations in order to be better able to make comparisons. The secretions of each donor male were randomly assigned to and incubated at a single temperature treatment (cold or hot), so swabs of 10 to 12 individuals from each population were used under each temperature. One of the swabs extracted from each donor individual was incubated for a short period of 1 min (control), and the other swab was incubated at the same temperature treatment for a long period of 4 hr. Each of the swabs was tested with sets of three different responding conspecific males coming from the pool of 22 to 24 males of the same population as the donor male. This paired design allowed us to look for differences in mean male response to femoral secretions before (short) and after (long) incubation at either hot or cold temperatures.

### 2.5 Chemosensory response trials

Behavioral trials were performed between 9:00 and 13:00 hr, when males were fully active. Males were allowed to bask in their terraria for 1 hr before the trials. We attached the cotton swab with the stimuli to a long stick of 40 cm. We then slowly approached a lizard's home container and slowly moved the stimuli to a position 2 cm anterior to the male snout. Once in that position, we recorded two measures of detectability: (1) how long (in seconds) it took the responding male to detect and start to tongue flick the swab (i.e., tongue flick LT), (2) the number of tongue flicks (TF) directed toward the swab in a one‐minute period following the first tongue flick. Shorter LTs and high tongue‐flick rates are indicators of greater persistency and detectability of the secretion. Each of the swabs from each donor male was used in tests with three different responding males, exposing the secretions obtained from one male to six different conspecific in total (Figure 1). The same person recorded the behavior of all males, blind to the treatment, and population of each individual.

**Figure 2:**
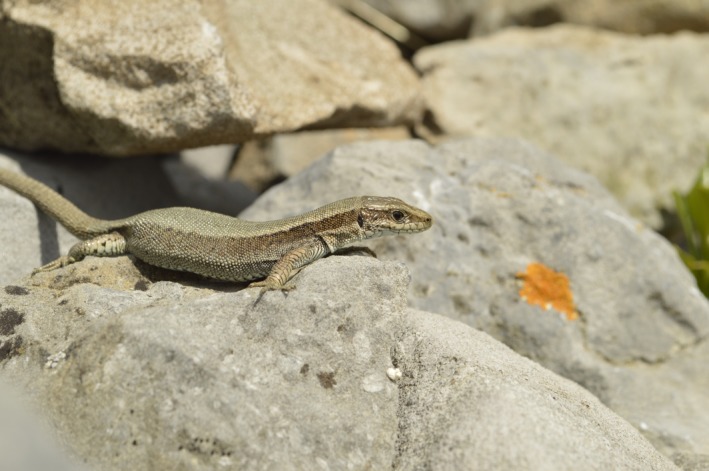
Female of the mountain lacertid lizard *Iberolacerta bonalli* in the rocky habitats of Pyrenees

### 2.6 Statistical analyses

For use in statistical analyses, we calculated the mean latency time (LT) and mean number of tongue flicks (TF) directed toward each swab by the three responding males, for both the short‐ and long‐incubation periods. We used generalized linear mixed models (GLMM) to test for differences in LT and TF between high‐ and low‐altitude populations of each species. Treatment (cold or hot), population altitude (high or low), and incubation time (short or long exposure to the corresponding temperature) were included as fixed factors. We also included all two‐way and three‐way interactions involving the temperature treatment, population altitude, and time. We included the donor individual as a random effect to control for individual variation in the response to their femoral secretions, and we specified a Poisson error distribution. When necessary we corrected for overdispersion by including a randomly assigned individual number (from 1 to *n*) as a random effect (Harrison, 2014). Pairwise comparisons were planned using Tukey's honestly significant difference tests. In the cases that a significant three‐way interaction was detected, we ran a separate model for each of the populations to determine the variables driving the significant interactions. In these models, treatment and time were included as fixed factors, and the individual as a random effect. Our main aim was to test for population differences in changes in signal efficacy before and after the temperature treatment (i.e., a three‐way interaction). However, when no three‐way interaction was detected, we performed sequential model reduction using likelihood ratio tests with a chi‐square error distribution to determine which predictor variables (if any) explained the variation in our data (see full model results in the Tables S1–S6).

To test for among species differences in LT and TF within each altitude population, we followed the same procedure as explained above. In this case, each of the GLMMs included treatment (hot or cold), incubation time (short or long exposure), species, and the interaction between them as fixed factors. All the models were run in R 3.2.2.

## 3 RESULTS

### 3.1 Within‐species differences between high‐ and low‐altitude populations

We found that the loss of signal efficacy in relation to temperature and altitude depended on the species in question (see below).

#### 3.1.1 *Podarcis muralis*

**Figure 3:**
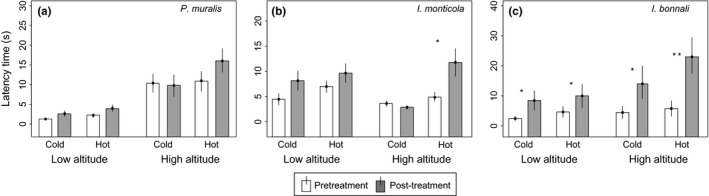
Latency time (mean + *SE*) in relation to population altitude, incubation time, and temperature treatment. Short exposure is shown in white. Long exposure is shown in gray. Significant comparisons (short vs. long exposure) are marked with an asterisk. Two asterisks show *p *<* *.001 in a Tukey's test

**Figure 4:**
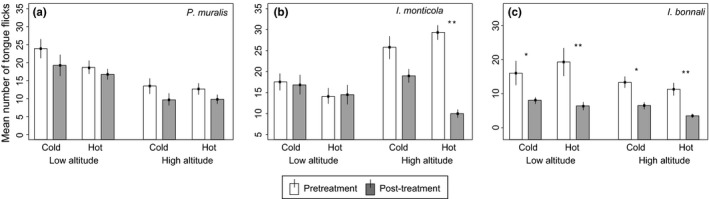
Mean number of tongue flicks (mean + *SE*) in relation to population altitude, incubation time, and temperature treatment. Short exposure is shown in white. Long exposure is shown in gray. Significant comparisons (short vs. long exposure) are marked with an asterisk. Two asterisks show *p *<* *.001 in a Tukey's test

In the case of the ubiquitous species, *P. muralis*, we found that the behavioral responses (LT and TF) elicited by secretions were not dependent on the three‐way interaction between population altitude, temperature treatment, and time (treatment*altitude*time − LT: estimate ± *SE* = 0.949 ± 0.816, *z* = 1.163, *p *=* *.244, Figure 3a; TF: estimate ±*SE* = −0.045 ± 0.322, *z* = −0.143, *p *=* *.886, Figure 4a, Table 1) indicating that the potential effects of temperature on the secretions from different altitudes, if any, were not different enough to provoke a different behavioral response.

**Table 1:** Effects of population altitude, temperature treatment and incubation time (short or long exposure) and their interactions on latency time, and number of tongue flicks in *Podarcis muralis* (results from GLMM)

Factors	Latency time				Number of tongue flicks			
	Estimate	*SE*	*z*	*p*	Estimate	*SE*	*z*	*p*
Intercept	1.794	0.250	7.164	**<.001**	2.176	0.132	16.524	**<.001**
Treatment	0.710	0.337	2.109	**.035**	0.034	0.182	0.189	.849
Altitude	−1.142	0.405	−2.820	**.005**	0.696	0.185	3.766	**<.001**
Time	0.168	0.348	0.483	.628	0.334	0.163	2.053	**.040**
Treatment*altitude	−0.252	0.550	−0.458	.647	−0.138	0.259	−0.533	.594
Treatment*time	−0.724	0.479	−1.512	.130	−0.075	0.226	−0.333	.739
Altitude*time	−0.934	0.605	−1.545	.122	−0.104	0.228	−0.455	.649
Treatment*altitude*time	0.949	0.816	1.163	.225	−0.046	0.322	−0.143	.886

Two and three‐way interactions between our variables are indicated with a *. Significant values are in bold.

In addition, we found that secretions from the low‐altitude population were more rapidly detected (shorter LT) and were more effective (higher TF rates) than those from the high‐altitude population (altitude − LT: estimate ± *SE* = −1.142 ± 0.405, *z* = −2.820, *p *=* *.005; TF: estimate ± *SE* = 0.696 ± 0.185, *z* = 3.766, *p *<* *.001). In general, secretions were less effective (TF) after being incubated for 4 h compared to 1 min (time: estimate ± *SE* =  .334 ± 0.163, *z* = 2.053, *p *=* *.040), but the LT was unaffected by incubation time (time: estimate ± *SE* = 0.168 ± 0.348, *z* = 0.483, *p *=* *.629). Latency times were, in general, longer after the exposure to hot temperatures (treatment: estimate ± *SE* = 0.710 ± 0.337, *z* = 2.109, *p *=* *.035), but no effect of the temperature was detected in the number of TFs (treatment: estimate ± *SE* = 0.034 ± 0.182, *z* = 0.189, *p *=* *.849). Sequential model reduction showed similar results (Table S1), suggesting these results are robust to the model used.

#### 3.1.2 *Iberolacerta monticola*

In contrast, for *I. monticola*, one of the mountain species, we found that the behavioral responses (LT and TF) elicited by secretions were dependent on the three‐way interaction between population altitude, temperature treatment, and incubation time (treatment*altitude*time − LT: estimate ± *SE* = −0.830 ± 0.289, *z* = −2.869, *p *=* *.004, Figure 3b; TF: estimate ± *SE* = −0.830 ± 0.289, *z* = −2.869, *p *=* *.004, Figure 4b, Table 2) indicating that the effect of being incubated at high and low temperatures on secretions from different altitudes was enough to elicit different behavioral responses. To investigate these differences further, we analyzed each altitude separately. For the high‐altitude population, secretions incubated at hot rather than cold temperatures for 4 hr had significantly longer LT than when the secretion was only exposed for the 1‐min control period (treatment*time: estimate ± *SE* = −1.120 ± 0.329, *z* = −3.402, *p *<* *.001, Table 3). This was not the case in the low‐altitude population, which showed no two‐way interaction (treatment*time: estimate ± *SE* = 0.274 ± 0.219, *z* = 1.251, *p *=* *.210, Table 3) but did show an overall effect of incubation time (time: estimate ± *SE* = −0.603 ± 0.161, *z* = −3.735, *p *<* *.001). A similar pattern was seen for the number of TFs elicited by the secretions. For the high‐altitude population, secretions incubated at hot rather than cold temperatures for 4 hr elicited significantly fewer TFs than when the secretion was only exposed for the 1‐min control period (treatment*time: estimate ± *SE* = 0.750 ± 0.161, *z* = 4.64, *p *<* *.001). For the low population, however, we found no significant two‐way interaction (treatment*time: estimate ± *SE* = −0.065 ± 0.146, *z* = −0.449, *p* = .654), and in this case, opposite to the high altitude population, we did not find an overall significant effect of incubation time (time: estimate ± *SE* = 0.040 ± 0.094, *z* = 0.428, *p* = .669).

**Table 2:** Effects of population altitude, temperature treatment and incubation time (short or long exposure) and their interactions in latency time (LT), and number of tongue flicks in *Iberolacerta monticola* (results from GLMM)

Factors	LT				Number of tongue flicks			
	Estimate	*SE*	*z*	*p*	Estimate	*SE*	*z*	*p*
Intercept	1.293	0.283	4.562	**<.001**	2.807	0.138	20.36	**<.001**
Treatment	1.015	0.334	3.036	**.002**	−0.543	0.187	−2.901	**.004**
Altitude	0.598	0.309	1.935	.053	−0.042	0.1543	−0.276	.782
Time	0.235	0.326	0.720	.471	0.297	0.149	1.985	**.047**
Treatment*altitude	−0.717	0.408	−1.760	.078	0.402	0.232	1.734	.083
Treatment*time	−1.07	0.408	−2.620	**.009**	0.765	0.217	3.531	**<.001**
Altitude*time	−0.829	0.392	−2.115	**.034**	−0.247	0.196	−1.262	.206
Treatment*altitude*time	1.358	0.509	2.665	**.008**	−0.831	0.289	−2.869	**.004**

Two and three‐way interactions between our variables are indicated with a *. Significant values are in bold.

**Table 3:** Effects of temperature treatment and incubation time (short or long exposure) and their interactions in latency time (LT) and number of tongue flicks in the low‐ and high‐altitude populations of *Iberolacerta monticola* (results from GLMM)

Factors	LT				Number of tongue flicks			
	Estimate	*SE*	*z*	*p*	Estimate	*SE*	*z*	*p*
Low‐altitude population								
Intercept	1.933	0.187	10.324	**<.001**	2.771	0.118	23.388	**<.001**
Treatment	0.213	0.269	0.789	.429	−0.163	0.177	−0.919	.358
Time	−0.603	0.161	−3.735	**<.001**	0.040	0.094	0.428	.669
Treatment*time	0.274	0.219	1.251	.211	−0.066	0.146	−0.449	.654
High‐altitude population								
Intercept	1.015	0.242	4.188	**<.001**	2.944	0.081	36.30	**<.001**
Treatment	1.382	0.287	4.822	**<.001**	−0.619	0.132	−4.69	**<.001**
Time	0.232	0.277	0.836	.403	0.304	0.107	2.84	**.004**
Treatment*time	−1.12	0.329	−3.402	**<.001**	0.75	0.161	4.64	**<.001**

Two and three‐way interactions between our variables are indicated with a *. Significant values are in bold.

#### 3.1.3 *Iberolacerta bonnali*

Like for *P. muralis*, in the Pyrenean mountain species, *I. bonnali*, we found no evidence that the behavioral responses (LT and TF) elicited by secretions were dependent on the three‐way interaction between population altitude temperature treatment and incubation time (treatment*altitude*time − LT: estimate ± *SE* = 0.785 ± 0.633, *z* = 1.239, *p *=* *.215, Figure 3c; TF: estimate ± *SE* = −0.001 ± 0.385, *z* = −0.004, *p *=* *.997, Figure 4c, Table 4) indicating that the effect of temperature on the secretions from different altitudes, if any, was not different enough to elicit different behavioral response.

**Table 4:** Effects of population altitude, temperature treatment and incubation time (short or long exposure) and their interactions in latency time (LT), and number of tongue flicks in *Iberolacerta bonnali* (results from GLMM)

Factors	LT				Number of tongue flicks			
	Estimate	*SE*	*z*	*p*	Estimate	*SE*	*z*	*p*
Intercept	2.258	0.229	9.859	**<.001**	1.939	0.169	11.432	**<.001**
Treatment	0.594	0.318	1.865	.062	−0.659	0.255	−2.585	**.009**
Altitude	−0.437	0.32	−1.364	.172	−0.008	0.234	−0.035	.972
Time	−1.136	0.297	−3.824	**<.001**	0.719	0.179	4.026	**<.001**
Treatment*altitude	−0.388	0.481	−0.807	.419	0.501	0.357	1.401	.161
Treatment*time	−0.329	0.418	−0.789	.430	0.415	0.279	1.489	.136
Altitude*time	−0.046	0.459	−0.100	.920	−0.102	0.257	−0.400	.689
Treatment*altitude*time	0.785	0.634	1.239	.215	−0.001	0.385	−0.004	.997

Two and three‐way interactions between our variables are indicated with a *. Significant values are in bold.

In addition, we found that, in general, secretions took longer to be detected (LT) and were less effective (TF) after being incubated for 4 hr compared to 1 min (time – LT: estimate ± *SE* = −1.136 ± 0.297, *z* = −3.824, *p *<* *.001; TF: estimate ± *SE* = 0.719 ± 0.179, *z* = 4.026, *p *<* *.001). When we ran the reduced models, the temperature treatment and time incubated became significant and we found that secretions from the low‐altitude population and cold treatment were detected more rapidly (LT – altitude: estimate ± *SE* = −0.482 ± 0.177, *z* = −2.714, *p *=* *.007; treatment: estimate ± *SE* = 0.430 ± 0.197, *z* = 2.184, *p *=* *.029) but were not more effective (TF – altitude: estimate ± *SE* = 0.186 ± 0.133, *z* = 1.396, *p *=* *.163; treatment: estimate ± *SE* = −0.169 ± 0.131, *z* = −1.287, *p *=* *.198) than those from the high‐altitude population and hotter treatment (Table S2).

### 3.2 Differences among species within low‐ and high‐altitude populations

We found no significant behavioral differences in LT or TF between the three species before and after incubation of secretions at hot and cold temperatures, in either the low (treatment*species*time − LT: estimate ± *SE* = 0.213, ±0.617, *z* = −0.346, *p *=* *.729; TF: estimate ± *SE *= −0.531 ± 0.327, *z* = −1.624, *p *=* *0.104, Table S3) or the high‐altitude populations (treatment*species*time: estimate ± *SE* = −1.146 ± 0.748, *z* = −1.531, *p *=* *.125; TF: estimate ± *SE* = −0.229 ± 0.368, *z* = −0.624, *p *=* *.532, Table S5). This indicated that within the low and high altitudes, the effect of temperature on secretions was not strong enough to elicit different behavioral responses.

However, we did find other species differences in behavioral responses to femoral secretions within each altitude. For the low‐altitude populations, *P. muralis* had shorter LT (species: χ^2^ = 63.99, *df* = 2, *p *<* *.001) and higher TF (species: species: χ^2^ = 43.17, *df* = 2, *p *<* *.001) than the two mountain species (all Tukey's tests *p* = <.001). In addition, for TF, we found a two‐way interaction between the incubation time and the species (time*species: χ^2^ = 25.14, *df* = 2, *p *<* *.001). Results from the reduced models are shown in the Table S4.

Importantly, in the high‐altitude populations, we found a two‐way interaction between the species and the incubation time (time*species: χ^2^ = 13.33, *df* = 2, *p *=* *.001). Only *I. bonnali* showed an increase in LT with increased incubation time (Tukey's tests *p *<* *.001). We also found that, overall, the increase in LT observed after the treatment was stronger when the secretions were exposed to hot temperatures (time*treatment: χ^2^ = 8.03, *df* = 2, *p *=* *.001). We found a two‐way interaction between the species and the time of exposure of secretions (time*species: χ^2^ = 25.54, *df* = 2, *p *<* *.001), such that both *Iberolacerta* species reduced the number of TF as incubation time increased (both Tukey's tests *p *<* *.001) (results from the reduced model in the Table S6).

## 4 DISCUSSION

We predicted that the reduction in signal detectability and/or efficacy due to increased temperatures would be stronger for species restricted to mountain regions and, within each species, for populations inhabiting higher altitudes. In support of this, our results showed that an increase in temperature may disrupt the sensory ecology of some lizard species, potentially altering sexual selection and social behavior, but that the extent of these detrimental effects are likely to depend on the environmental conditions to which the species/population is adapted to.

### 4.1 Effects of the increase in temperature in each species

In the ubiquitous species, *P. muralis*, chemical signals from low‐altitude populations are, irrespective of temperature, more effective at eliciting a response than those from high‐altitude populations. As the males that donated the signals, as well as the responding males used in behavioral trials, were both from the same population it is not possible to disentangle whether these differences between altitudes are a result of differences in signal transmission or signal detection. This result could suggest that the populations differ in their use of or reliance on chemical signals perhaps due to differences in their social system (Brown, 1995; Edsman, 1991). The fact that temperature did not interact with time or population altitude to influence male behavioral responses toward the secretions suggests that *P. muralis* is may be resilient to environmental temperature changes, at least in the range of temperatures tested here.

The chemical signals of the Pyrenean mountain species, *I. bonnali*, showed a strong overall effect of incubation time regardless of altitude or temperature. Given that this is the most altitudinally restricted species and the one found at the highest altitude (see Section 2), it could be that both the low‐ and high‐altitude populations of this species have chemical signals that are adapted to even colder temperatures than those tested in this experiment. If *I. bonnali* secretions are adapted to cold temperatures, our results suggest that an increase in environmental temperature would have a more detrimental effect on this species than in the other two. Alternatively, as this species is not very territorial (Martínez‐Rica, 1977), chemical communication might not be as important as in other lizards, so the male scent marks would not need to last for long time. If this is the case, males could use chemical signaling to communicate when encountering an interspecific, but not for long‐term territorial scent marking.

Interestingly, the only species that showed the predicted altitude‐dependent response of chemical signals to differences in incubation temperature, *I. monticola*, was also the species with the intermediate altitudinal distribution and intermediate geographic range. This finding could reflect the possibility that this species is the only one tested for which our experimental temperatures span those in which the lizards naturally deposit their femoral secretions. To gain a better understanding of the causes of differences in response by the different species and populations further, studies on the thermal ecology of these species in their natural habitat would be useful.

### 4.2 Sensitivity of chemical signals to environmental change

When interpreting our results it is important to remember the limitations of our experiment. One limitation is that we have not tested for chemical changes in the composition of the secretions with temperature, as such evidence for a reduction in signal durability and detectability is based on the behavioral changes of the receiver males. As our temperature treatment was applied to the femoral secretions themselves and not the signaler or receiver male, and because the secretions of all three species have similar chemical properties, we believe it is likely that changes in male responses to secretions that had been incubated at different temperatures are due to differences in chemical composition of the scent marks and how they break down in different temperatures. A further limitation relates to the fact that the durability and detectability of scent marks in the wild depend on the combination of temperature, humidity and the substrate of deposition, and on the complexity of the factors related to lizard behavior (e.g., mating system). Therefore, disentangling what is currently happening in nature based in our experiment is difficult. Finally, the species used in our experiment differ in many ways, and our experimental design does not allow us to tease apart which ecological factors underlie the species‐specific behavioral responses we see here. Below we highlight several possible explanations that could be related to the differences in the detectability of signals of different species and populations. However, future comparative studies would be useful for teasing apart the combined factors that could be behind these differences (e.g., ecology, diet, and thermal range).

One possible explanation for our results is that the breadth of geographic distribution and intrinsic factors, such as the preferred thermal range, that influences the thermal microhabitat that each species inhabits, might influence adaptation of chemical signals to the environment. Therefore, species inhabiting a wide variety of environments might be adapted to broad environmental conditions, so they might be better able to cope with environmental variation. Accordingly, the behavioral response of *P. muralis* males (the species with a broad geographical distribution) to the femoral secretions of conspecifics from high‐ and low‐altitude populations was not dependent on the incubation temperature of the chemical signals. Thermal tolerance breadths of ectotherms inhabiting places with greater seasonal thermal variations (such as *P. muralis*) are wider than those experiencing more restricted thermal ranges (such as *I. monticola* and *I. bonalli,* whose breeding season is restricted to a short period with quite stable weather) (Sunday, Bates, & Dulvy, 2011). In addition, *P. muralis* has a broad geographic range with high levels of gene flow between locations which could impede adaptation to local environmental conditions (Salvador, 2014) and contribute to the broad thermal tolerances of this species (Claussen, Townsley, & Bausch, 1990; Salvador, 2014). If this is the case, the secretions of high‐ and low‐altitude populations of this species are likely to be effective in a broad range of thermal environments.

Restricted ecological requirements and local adaptation of some species, such as the mountain lizard species we study here, might constrain the efficacy of chemical signals in the face of environmental changes. The compounds and their proportions present in lizard secretions vary across populations and species, possibly as a response to specific habitats and climate regimes (Escobar et al., 2003; Martín et al., 2017). For instance, the secretions of lizards from warm areas are richer in compounds that have lower volatility and higher stability than secretions from species adapted to colder temperatures (Alberts, 1992; Apps et al., 2015). Similarly, the restricted distribution and the isolation of the mountain populations studied here might have undergone local adaptation to improve the efficacy of signals in the specific environmental conditions (Martín et al., 2015). This could explain the strong effect of incubation time in the reduction of the efficacy of the chemical signals in both cold and hot treatments of *I. bonnali*. This suggests that chemical compounds responsible for the behavioral response evaporate and degrade over time (Epple, Alveario, Golob, & Smith, 1980). However, the reduction in signal efficacy did not depend on the incubation temperature of the secretions. This might reflect a high tolerance of *I. bonnali* to temperature changes. This potential resistance to increased temperature has been previously suggested, as this species has broader thermal ranges and more effective thermoregulation ability than other species of the same genus (Ortega, Mencía, & Pérez‐Mellado, 2016).

In *I. monticola*, the loss in signal efficacy over time was stronger at higher temperatures but only in the high‐altitude population. One of the processes that might drive this finding is the higher evaporation ratios of the chemical compounds on the high‐altitude population (McDonough, Brown, & Aller, 1989), which probably depends on the specific composition of the chemical signals and the environmental conditions where animals come from. This claim could be easily tested in the laboratory by analyzing the chemical composition of secretions from different populations, and exploring the degradation of such compounds over time, at different temperatures. The isolation and specific climatic conditions of the high‐altitude population might have undergone local adaptation of secretions for a chemical composition more sensitive to temperature rise (Martín et al., 2015, 2017). In this situation, adaptive responses in the chemical composition of the secretions or in the receiver vomeronasal system (i.e., systems more sensitive to temperature‐degraded scent marks) of *I. monticola* might be critical to avoid a reduction in signaling efficacy in the face of rapid environmental change. However, although evolutionary adaptation over short time spans in response to anthropic disturbances occurs in a variety of species (Bradshaw & Holzapfel, 2006), and rapid evolutionary adaptation is unlikely in the majority of species (Sih, Ferrari, & Harris, 2011). Critically, specialist species like *I. monticola* have been proposed to be particularly vulnerable due to limiting evolutionary potential (Williams et al., 2008).

### 4.3 Consequences of the disruption of chemical communication in lizards

The disruption of chemical communication could have important ecological, evolutionary, and conservation consequences in lizards. Scent marks released by males are honest signals of the quality of the signaler as a potential mate for females and as a competitor for other males (Mason & Parker, 2010). Previous studies have shown a reduction in the efficacy of sexual signals of many taxa due to human‐induced environmental change (reviewed in Lürling & Scheffer, 2007). For example, massive algae growth related to eutrophication increased the time and energy spent by *Gasterosteus aculeatus* males in courtship, while at the same time, relaxed the strength of selection in the male red courtship coloration (Candolin, Salesto, & Evers, 2007). Similarly, increased volatility and reduced permanency of scent marks may decrease the information that receivers can gain from chemical signals, reducing their ability to assess male quality (Martín & López, 2012). In such cases, females would approach male territories indiscriminately, potentially relaxing the strength of sexual selection on chemical signals and the male qualities that they advertise. Likewise, male competitors may engage in more escalated fights (Carazo et al., 2007), increasing costs associated with potential injury, and time and energy expenditure that reduce the resources they can allocate to other important life history traits and behaviors (Cooper, 1999). This could potentially affect population viability (e.g., if females are no longer able to choose high quality males) and/or favors the use of alternative communication systems (e.g., visual) (Fox & Shipman, 2003).

It is important to remember that in our experiment, the incubation treatments (i.e., time and temperature) were applied to the femoral secretions of males from each species/population, but that the behavioral trials took place in a common environment. As such, any effects of incubation time or temperature treatment on the behaviors that we measured must be the direct result of changes to the femoral secretions themselves and not due to changes in the behavior of either the signal transmitter or receiver. However, our experiment does not allow us to explore which components are responsible for eliciting a behavioral response, or whether these compounds are the same across the species and populations. In the wild, individuals could mitigate the effects of temperature on chemical signals by changing their behavior (e.g., altering location or frequency of secretion deposition). However, short‐term adjustments can be energetically costly, and some species may lack the physiological capacity to modify their behavior (Oberweger & Goller, 2001). Future studies might benefit from investigating under what conditions such behavioral compensation might occur.

## 5 CONCLUSIONS

In conclusion, our results suggest that the sensitivity of chemical communication of lizards to differences in temperature depends on the specific ecological characteristics of species and populations. Specialist species with stricter ecological requirements, such as mountain species, are more prone to be affected by increased temperature. Further studies comparing additional species and populations are needed to determine whether the results presented here apply generally to lizards and whether ecological requirements of species and specific adaptations of populations to their microhabitat increase or reduce the potential negative effects of increased temperatures. Behavioral studies like the present one, in combination with physiological and phylogenetic approaches, are critical to understand how specific environmental changes can affect the ecology and hence, the conservation of endangered species.

## CONFLICT OF INTEREST

None declared.

## AUTHOR CONTRIBUTIONS

MIG, CC, and JM designed the experiment. MIG and CC collected the data. MIG and MH analyzed the data and interpreted the results. MIG, MH, JM, and CC drafted the manuscript.

## References

[ece33646-bib-0001] Alberts, A. (1992). Constraints on the design of chemical communication systems in terrestrial vertebrates. The American Naturalist, 139, S62–S89. https://doi.org/10.1086/285305

[ece33646-bib-0002] Apps, P. J. , Weldon, P. J. , & Kramer, M. (2015). Chemical signals in terrestrial vertebrates: search for design features. Natural Products Reports, 32(7), 1131–1153. https://doi.org/10.1039/C5NP00029G 10.1039/c5np00029g 26100000

[ece33646-bib-0003] Billingham, Z. D. , Chapple, D. G. , Sunnucks, P. , & Wong, B. B. M. (2009). Chemical cues and group association preferences in a subsocial cockroach, *Panesthia australis* . Australian Journal of Zoology, 57(6), 385–390. https://doi.org/10.1071/ZO09066

[ece33646-bib-0004] Blackburn, T. , Lockwood, J. , & Cassey, P. (2009). Avian invasions: The ecology and evolution of exotic birds. Oxford: Oxford University https://doi.org/10.1093/acprof:oso/9780199232543.001.0001

[ece33646-bib-0005] Bradshaw, W. , & Holzapfel, C. (2006). Evolutionary response to rapid climate change. Science, 312, 1477–1478. https://doi.org/10.1126/science.1127000 16763134 10.1126/science.1127000

[ece33646-bib-0006] Brown, J. (1995). Macroecology. Chicago, IL: University of Chicago Press.

[ece33646-bib-0007] Brown, R. M. , Gist, D. H. , & Taylor, D. H. (1995). Home range ecology of an introduced population of the European Wall Lizard Podarcis muralis (Lacertilia; Lacertidae) in Cincinnati, Ohio. The American Midland Naturalist, 133(2), 344–359. https://doi.org/10.2307/2426399

[ece33646-bib-0008] Buckley, L. B. (2010). The range implications of lizard traits in changing environments. Global Ecology and Biogeography, 19(4), 452–464.

[ece33646-bib-0009] Candolin, U. , Salesto, T. , & Evers, M. (2007). Changed environmental conditions weaken sexual selection in sticklebacks. Journal of Evolutionary Biology, 20(1), 233–239. https://doi.org/10.1111/j.1420-9101.2006.01207.x 17210016 10.1111/j.1420-9101.2006.01207.x

[ece33646-bib-0010] Carazo, P. , Font, E. , & Desfilis, E. (2007). Chemosensory assessment of rival competitive ability and scent‐mark function in a lizard, *Podarcis hispanica* . Animal Behaviour, 74(4), 895–902. https://doi.org/10.1016/j.anbehav.2007.02.011

[ece33646-bib-0011] Carazo, P. , Font, E. , & Desfilis, E. (2008). Beyond, “nasty neighbours” and “dear enemies”? Individual recognition by scent marks in a lizard (*Podarcis hispanica*). Animal Behavior, 76(6), 1953–1963. https://doi.org/10.1016/j.anbehav.2008.08.018

[ece33646-bib-0012] Cassey, P. , Blackburn, T. M. , Sol, D. , Duncan, R. P. , & Lockwood, J. L. (2004). Global patterns of introduction effort and establishment success in birds. Proceedings of the Royal Society B‐Biological Sciences, 271, S405–S408. https://doi.org/10.1098/rsbl.2004.0199 10.1098/rsbl.2004.0199 PMC1810115 15801588

[ece33646-bib-0013] Claussen, D. L. , Townsley, M. D. , & Bausch, R. G. (1990). Supercooling and freeze‐tolerance in the European wall lizard, *Podarcis muralis*, with a revisional history of the discovery of freeze‐tolerance in vertebrates. Journal of Comparative Physiology B, 160(2), 137–143. https://doi.org/10.1007/BF00300945

[ece33646-bib-0014] Cooper, W. E. (1999). Tradeoffs between courtship, fighting, and antipredatory behavior by a lizard, *Eumeces laticeps* . Behavioral Ecology and Sociobiology, 47(1–2), 54–59. https://doi.org/10.1007/s002650050649

[ece33646-bib-0015] Cross, H. B. , Blumstein, D. T. , & Rosell, F. (2013). Do marmots display a “dear enemy phenomenon” in response to anal gland secretions? Journal of Zoology, 289(3), 189–195. https://doi.org/10.1111/j.1469-7998.2012.00975.x

[ece33646-bib-0016] Edsman, L. (1991). Strategies of territory take‐overs in wall lizards. In Abstracts of the Sixth Ordinary General Meeting of the Societas Europaea Herpetologica (p. 28). Budapest.

[ece33646-bib-0017] Endler, J. A. (1992). Signals, signal conditions, and the direction of evolution. The American Naturalist, 139, S125–S153. https://doi.org/10.1086/285308

[ece33646-bib-0018] Engström‐Öst, J. , & Candolin, U. (2006). Human‐induced water turbidity alters selection on sexual displays in sticklebacks. Behavioral Ecology, 18(2), 393–398.

[ece33646-bib-0019] Epple, G. , Alveario, M. C. , Golob, N. F. , & Smith, A. B. (1980). Stability ad attractiveness related to age of scent marks of saddle back tamarins (*Saguinus fuscicollis*). Journal of Chemical Ecology, 6, 735–748. https://doi.org/10.1007/BF00990398

[ece33646-bib-0020] Escobar, C. , Labra, A. , Niemeyer, H. M. , & Escobar, C. (2003). Chemical composition of precloacal secretions of two *Liolaemus fabiani* populations: Are they different? Journal of Chemical Ecology, 29(3), 629–638. https://doi.org/10.1023/A:1022858919037 12757324 10.1023/a:1022858919037

[ece33646-bib-0021] Fabian, N. J. , Albright, L. B. , Gerlach, G. , Fisher, H. S. , & Rosenthal, G. G. (2007). Humic acid interferes with species recognition in zebrafish (*Danio rerio*). Journal of Chemical Ecology, 33(11), 2090–2096. https://doi.org/10.1007/s10886-007-9377-z 17952510 10.1007/s10886-007-9377-z

[ece33646-bib-0022] Foden, W. B. , Butchart, S. H. M. , Stuart, S. N. , Vié, J. C. , Akçakaya, H. R. , Angulo, A. , … Mace, G. M. (2013). Identifying the world's most climate change vulnerable species: A systematic trait‐based assessment of all birds, amphibians and corals. PLoS ONE, 8(6), e65427 https://doi.org/10.1371/journal.pone.0065427 23950785 10.1371/journal.pone.0065427 PMC3680427

[ece33646-bib-0023] Fox, S. F. , & Shipman, P. A. (2003). Social behavior at high and low elevations: Environmental release and phylogenetic effects in *Liolaemus* In Fox F., McCoy J., & Baird T. (Eds.), Lizard social behavior (pp. 310–355). New York, NY: John Hopkins University Press.

[ece33646-bib-0024] Hamer, A. J. , & McDonnell, M. J. (2008). Amphibian ecology and conservation in the urbanising world: A review. Biological Conservation, 141(10), 2432–2449. https://doi.org/10.1016/j.biocon.2008.07.020

[ece33646-bib-0025] Harrison, X. A. (2014). Using observation‐level random effects to model overdispersion in count data in ecology and evolution. PeerJ, 2, e616 https://doi.org/10.7717/peerj.616 25320683 10.7717/peerj.616 PMC4194460

[ece33646-bib-0027] Iglesias‐Carrasco, M. , Head, M. L. , Jennions, M. D. , & Cabido, C. (2017). Secondary compounds from exotic tree plantations change female mating preferences in the palmate newt (*Lissotriton helveticus*). Journal of Evolutionary Biology, 30, 1788–1795. https://doi.org/10.1111/jeb.13091 28419678 10.1111/jeb.13091

[ece33646-bib-0028] Iglesias‐Carrasco, M. , Head, M. L. , Jennions, M. D. , Martín, J. , & Cabido, C. (2017). Leaf extracts from an exotic tree affect responses to chemical cues in the palmate newt, *Lissotriton helveticus* . Animal Behaviour, 127, 243–251. https://doi.org/10.1016/j.anbehav.2017.03.025

[ece33646-bib-0029] Komeza, N. , Fouillet, P. , Boulétreau, M. , & Delpuech, J. M. (2001). Modification, by the insecticide chlorpyrifos, of the behavioral response to kairomones of a parasitoid wasp, *Leptopilina boulardi* . Archives of Environmental Contamination and Toxicology, 41(4), 436–442. https://doi.org/10.1007/s002440010269 11598780 10.1007/s002440010269

[ece33646-bib-0030] Leal, M. , & Fleishman, L. J. (2004). Differences in visual signal design and detectability between allopatric populations of *Anolis* lizards. The American Naturalist, 163(1), 26–39. https://doi.org/10.1086/379794 10.1086/379794 14767834

[ece33646-bib-0031] Lemasson, A. , Mikus, M. A. , Blois‐Heulin, C. , & Lodé, T. (2013). Social partner discrimination based on sounds and scents in Asian small‐clawed otters (*Aonyx cinereus*). Naturwissenschaften, 100(3), 275–279. https://doi.org/10.1007/s00114-013-1022-9 23397263 10.1007/s00114-013-1022-9

[ece33646-bib-0032] Lürling, M. , & Scheffer, M. (2007). Info‐disruption: Pollution and the transfer of chemical information between organisms. Trends in Ecology & Evolution, 22(7), 374–379. https://doi.org/10.1016/j.tree.2007.04.002 17433848 10.1016/j.tree.2007.04.002

[ece33646-bib-0034] Martín, J. , Javier Zamora‐Camacho, F. , Reguera, S. , López, P. , & Moreno‐Rueda, G. (2017). Variations in chemical sexual signals of *Psammodromus algirus* lizards along an elevation gradient may reflect altitudinal variation in microclimatic conditions. The Science of Nature, 104(3–4), 16 https://doi.org/10.1007/s00114-017-1442-z 10.1007/s00114-017-1442-z 28251299

[ece33646-bib-0035] Martín, J. , & López, P. (2012). Supplementation of male pheromone on rock substrates attracts female rock lizards to the territories of males: A field experiment. PLoS ONE, 7(1), e30108 https://doi.org/10.1371/journal.pone.0030108 22253895 10.1371/journal.pone.0030108 PMC3258258

[ece33646-bib-0036] Martín, J. , & López, P. (2013). Effects of global warming on sensory ecology of rock lizards: Increased temperatures alter the efficacy of sexual chemical signals. Functional Ecology, 27(6), 1332–1340. https://doi.org/10.1111/1365-2435.12128

[ece33646-bib-0037] Martín, J. , & López, P. (2014). Pheromones and chemical communication in lizards In Rheubert J. L., Siegel D. S., & Trauth S. E. (Eds.), The reproductive biology and phylogeny of lizards and tuatara (pp. 43–77). Boca Ratón, FL: CRC Press.

[ece33646-bib-0038] Martín, J. , & López, P. (2015). Condition‐dependent chemosignals in reproductive behavior of lizards. Hormones and Behavior, 68, 14–24. https://doi.org/10.1016/j.yhbeh.2014.06.009 24952102 10.1016/j.yhbeh.2014.06.009

[ece33646-bib-0039] Martín, J. , Ortega, J. , & López, P. (2015). Interpopulational variations in sexual chemical signals of Iberian wall lizards may allow maximizing signal efficiency under different climatic conditions. PLoS ONE, 10(6), e0131492 https://doi.org/10.1371/journal.pone.0131492 26121693 10.1371/journal.pone.0131492 PMC4488078

[ece33646-bib-0040] Martínez‐Rica, J. P. (1977). Observaciones ecológicas de Lacerta monticola bonnali, Lantz en el Pirineo españo. Publicaciones del Centro Pirenaico de Biología Experimental, 8, 103–122.

[ece33646-bib-0041] Mason, R. T. , & Parker, M. R. (2010). Social behavior and pheromonal communication in reptiles. Journal of Comparative Physiology. A, Neuroethology, Sensory, Neural, and Behavioral Physiology, 196(10), 729–749. https://doi.org/10.1007/s00359-010-0551-3 10.1007/s00359-010-0551-3 20585786

[ece33646-bib-0042] McDonough, L. M. , Brown, D. F. , & Aller, W. C. (1989). Insect sex pheromones – Effect of temperature on evaporation rates of acetates from rubber septa. Journal of Chemical Ecology, 15(3), 779–790. https://doi.org/10.1007/BF01015176 24271883 10.1007/BF01015176

[ece33646-bib-0043] Moreira, P. L. , Almeida, A. P. , Delgado, H. , Salgueiro, O. , & Crespo, E. G. (1998). Bases para a Conservação da Lagartixa‐da‐montanha (Lacerta monticola). Lisboa: Instituto da Conservação da Natureza, Ministerio do Ambiente.

[ece33646-bib-0044] Nemeth, E. , & Brumm, H. (2010). Birds and anthropogenic noise: Are urban songs adaptive? The American Naturalist, 176(4), 465–475. https://doi.org/10.1086/656275 10.1086/656275 20712517

[ece33646-bib-0045] Nemeth, E. , Winkler, H. , & Dabelsteen, T. (2001). Differential degradation of antbird songs in a neotropical rainforest: Adaptation to perch height? Journal of the Acoustical Society of America, 110(6), 3263–3274. https://doi.org/10.1121/1.1420385 11785827 10.1121/1.1420385

[ece33646-bib-0046] Oberweger, K. , & Goller, F. (2001). The metabolic cost of birdsong production. Journal of Experimental Biology, 204, 3379–3388. 11606611 10.1242/jeb.204.19.3379

[ece33646-bib-0047] Olsson, M. , Madsen, T. , Nordby, J. , Wapstra, E. , Ujvari, B. , & Wittsell, H. (2003). Major histocompatibility complex and mate choice in sand lizards. Proceedings of the Royal Society B‐Biological Sciences, 270, S254–S256. https://doi.org/10.1098/rsbl.2003.0079 10.1098/rsbl.2003.0079 PMC1809963 14667398

[ece33646-bib-0048] Ortega, Z. , Mencía, A. , & Pérez‐Mellado, V. (2016). The peak of thermoregulation effectiveness: Thermal biology of the Pyrenean rock lizard, *Iberolacerta bonnali* (Squamata, Lacertidae). Journal of Thermal Biology, 56, 77–83. https://doi.org/10.1016/j.jtherbio.2016.01.005 26857980 10.1016/j.jtherbio.2016.01.005

[ece33646-bib-0049] Pérez‐Mellado, V. , Sá‐Sousa, P. , Marquez, R. , & Martínez‐Solano, I. (2009). Iberolacerta monticola. UCN Red List of Threatened Species.

[ece33646-bib-0050] Pleguezuelos, J. M. , & Villafranca, C. (1997). Distribución altitudinal de la herpetofauna ibérica In Pleguezuelos J. M. (Ed.), Distribución y biogeografía de los anfibios y reptiles en España y Portugal (pp. 321–341). Granada, Spain: Asociación Herpetológica Española y Universidad de Granada.

[ece33646-bib-0051] Pottier, G. (2001). Nouvelle donnée sur la limite occidentale de répartition du lézard des Pyrénées *Iberolacerta bonnali* (Lantz, 1927) (Sauna, Lacertidae). Bulletin de la Societe Herpetologique de France, 98, 5–9.

[ece33646-bib-0052] Salvador, A. (2014). *Podarcis muralis* (Laurenti, 1768) In Salvador A. (Ed.), Reptiles, 2a edición revisada y aumentada Fauna Ibérica (pp. 576–589). Madrid, Spain: Museo Nacional de Ciencias Naturales, Consejo Superior de Investigaciones Científicas.

[ece33646-bib-0054] Sherba, M. , Dunham, D. W. , & Harvey, H. H. (2000). Sublethal copper toxicity and food response in the freshwater crayfish *Cambarus bartonii* (Cambaridae, Decapoda, Crustacea). Ecotoxicology and Environmental Safety, 46(3), 329–333. https://doi.org/10.1006/eesa.1999.1910 10903830 10.1006/eesa.1999.1910

[ece33646-bib-0055] Sih, A. , Ferrari, M. C. O. , & Harris, D. J. (2011). Evolution and behavioural responses to human‐induced rapid environmental change. Evolutionary Applications, 4(2), 367–387. https://doi.org/10.1111/j.1752-4571.2010.00166.x 25567979 10.1111/j.1752-4571.2010.00166.x PMC3352552

[ece33646-bib-0056] Sunday, J. M. , Bates, A. E. , & Dulvy, N. K. (2011). Global analysis of thermal tolerance and latitude in ectotherms. Proceedings of the Royal Society of London B: Biological Sciences, 278(1713), 1823–1830. https://doi.org/10.1098/rspb.2010.1295 10.1098/rspb.2010.1295 PMC3097822 21106582

[ece33646-bib-0057] Wei, H. Y. , & Du, J. W. (2004). Sublethal effects of larval treatment with deltamethrin on moth sex pheromone communication system of the Asian corn borer, *Ostrinia furnacalis* . Pesticide Biochemistry and Physiology, 80(1), 12–20. https://doi.org/10.1016/j.pestbp.2004.05.001

[ece33646-bib-0058] Williams, S. E. , Shoo, L. P. , Isaac, J. L. , Hoffmann, A. A. , & Langham, G. (2008). Towards an integrated framework for assessing the vulnerability of species to climate change. PLoS Biology, 6(12), e325 https://doi.org/10.1371/journal.pbio.0060325 10.1371/journal.pbio.0060325 PMC2605927 19108608

[ece33646-bib-0059] Wolf, M. C. , & Moore, P. A. (2002). Effects of the herbicide metolachlor on the perception of chemical stimuli by *Orconectes rusticus* . Journal of the North American Benthological Society, 21(3), 457–467. https://doi.org/10.2307/1468482

[ece33646-bib-0060] Wyatt T. (Ed.) (2014). Pheromones and animal behaviour: Chemical signals and signatures. Cambridge: Cambridge University Press.

[ece33646-bib-0061] Zahavi, A. (1991). On the definition of sexual selection, Fisher's model, and the evolution of waste and of signals in general. Animal Behavior, 42(3), 501–503. https://doi.org/10.1016/S0003-3472(05)80052-1

