# Supplementary material for: Increased temperature disrupts chemical communication in some species but not others: The importance of local adaptation and distribution
Source: Ecol Evol. 2017 Dec 15;8(2):1031–42. doi: 10.1002/ece3.3646 (PMC5773306; doi:10.1002/ece3.3646)
Supplement: Supplementary file 1 [file ECE3-8-1031-s001.docx]

**Supplementary material for:**

**Increased temperature disrupts chemical communication in some species but not others: the importance of local adaptation and distribution**

Table S1. Results from the reduced models based on Likelihood ratio tests. Effect of the population altitude, temperature treatment and incubation time (short or long exposure) and their interactions on the latency time and tongue flicks in *P. muralis*.

| *Podarcis muralis* | | | | | | | | | | | | |
| --- | --- | --- | --- | --- | --- | --- | --- | --- | --- | --- | --- | --- |
|  | Latency time | | | | | | Number of tongue flicks | | | | | |
| **Reduced model** | **Estimate** | | **SE** | | **z** | **p** | **Estimate** | **SE** | **z** | | | **p** |
| Intercept | 2.020 | | 0.188 | | 10.737 | **< 0.001** | 2.267 | 0.096 | 23.489 | | | **< 0.001** |
| Treatment | 0.421 | | 0.196 | | 2.153 | **0.031** | -0.081 | 0.099 | -0.820 | | | 0.412 |
| Altitude | -1.476 | | 0.204 | | -7.231 | **< 0.001** | 0.561 | 0.099 | 5.636 | | | **< 0.001** |
| Time | -0.363 | | 0.195 | | -1.858 | 0.063 | 0.234 | 0.081 | 2.885 | | | **0.004** |
| **Removed terms** | | ***X^2^*** | | **df** | | **p** | ***X^2^*** | | | **df** | **p** | |
| Treatment*time*altitude | | 1.353 | | 1 | | 0.245 | 0.020 | | | 1 | 0.886 | |
| Treatment*time | | 1.119 | | 1 | | 0.290 | 0.366 | | | 1 | 0.545 | |
| Time*altitude | | 1.046 | | 1 | | 0.306 | 0.583 | | | 1 | 0.445 | |
| Treatment*altitude | | 0.200 | | 1 | | 0.654 | 0.649 | | | 1 | 0.420 | |

Table S2. Results from the reduced models based on Likelihood ratio tests. Effect of the population altitude, temperature treatment and incubation time (short or long exposure) and their interactions on the latency time and tongue flicks in *I.bonnali*.

| *Iberolacerta bonnali* | | | | | | | | | | | |
| --- | --- | --- | --- | --- | --- | --- | --- | --- | --- | --- | --- |
|  | Latency time | | | | | | Number of tongue flicks | | | | |
| **Reduced model** | **Estimate** | **SE** | | **z** | **p** | | **Estimate** | **SE** | | **z** | **p** |
| Intercept | 2.273 | 0.185 | | 12.26 | **< 0.001** | | 1.735 | 0.133 | | 13.003 | **< 0.001** |
| Treatment | 0.43 | 0.197 | | 2.184 | **0.029** | | -0.169 | 0.131 | | -1.287 | 0.198 |
| Altitude | -0.482 | 0.177 | | -2.714 | **0.007** | | 0.186 | 0.133 | | 1.396 | 0.163 |
| Time | -1.144 | 0.16 | | -7.129 | **< 0.001** | | 0.852 | 0.102 | | 8.323 | **< 0.001** |
| **Removed terms** | ***X^2^*** | | **df** | | | **p** | ***X^2^*** | | **df** | | **p** |
| Treatment*time*altitude | 1.519 | | 1 | | | 0.217 | 0 | | 1 | | 0.997 |
| Treatment*time | 0.001 | | 1 | | | 0.968 | 3.883 | | 1 | | **0.049** |
| Treatment*altitude | 0.026 | | 1 | | | 0.872 | 3.449 | | 1 | | 0.063 |
| Time*altitude | 1.302 | | 1 | | | 0.254 | 0.289 | | 1 | | 0.591 |

S3. Effects of species, temperature treatment and incubation time (short or long exposure) and their interactions in latency time and number of tongue flicks in the low altitude populations of the three studied species (results from GLMM).

| Low altitude population (pooling together the three species studied) | | | | | | | | |
| --- | --- | --- | --- | --- | --- | --- | --- | --- |
|  | Latency time | | | | Number of tongue flicks | | | |
| **Factors** | **Estimate** | **SE** | **z** | **p** | **Estimate** | **SE** | **z** | **p** |
| Intercept | 1.851 | 0.203 | 9.106 | **< 0.001** | 2.083 | 0.153 | 13.624 | **< 0.001** |
| Treatment hot | 0.244 | 0.269 | 0.906 | 0.365 | -0.331 | 0.211 | -1.570 | 0.116 |
| Species *I. monticola* | 0.041 | 0.257 | 0.162 | 0.872 | 0.617 | 0.181 | 3.4 | **< 0.001** |
| Species *P. muralis* | -1.093 | 0.294 | -3.716 | **< 0.001** | 0.785 | 0.177 | 4.426 | **< 0.001** |
| Time (Short exposure) | -1.188 | 0.292 | -4.069 | **< 0.001** | 0.625 | 0.177 | 3.538 | **< 0.001** |
| Treat Hot * Sp. *I. monticola* | -0.010 | 0.395 | -0.026 | 0.979 | 0.242 | 0.282 | 0.856 | 0.392 |
| Treat Hot * Sp. *P. muralis* | 0.152 | 0.421 | 0.361 | 0.718 | 0.300 | 0.271 | 1.106 | 0.269 |
| Treat Hot * Time | 0.462 | 0.391 | 1.183 | 0.237 | 0.413 | 0.254 | 1.627 | 0.104 |
| Sp *I. monticola* * Time | 0.611 | 0.380 | 1.608 | 0.108 | -0.574 | 0.228 | -2.518 | **0.012** |
| Sp *P. muralis* * Time | 0.395 | 0.471 | 0.840 | 0.401 | -0.398 | 0.228 | -1.748 | 0.080 |
| Treat Hot * Sp. *I. monticola** Time | -0.196 | 0.521 | -0.277 | 0.706 | -0.479 | 0.334 | -1.434 | 0.151 |
| Treat. Hot * Sp. *P. muralis* * Time | -0.214 | 0.618 | -0.346 | 0.729 | -0.531 | 0.327 | -1.624 | 0.104 |

Table S4. Results from the reduced models based on Likelihood ratio tests. Effect of the species, temperature treatment and incubation time (short or long exposure) and their interactions on the latency time and tongue flicks in the low altitude populations of the three studied species.

| Low altitude population (pooling together the thee species studied) | | | | | | |
| --- | --- | --- | --- | --- | --- | --- |
|  | Latency time | | | Number of tongue flicks | | |
| **Reduced model** |  |  |  |  |  |  |
|  | ***X^2^*** | **df** | **p** | ***X^2^*** | **df** | **p** |
| Treatment | 9.693 | 1 | **0.002** | 1.206 | 1 | 0.272 |
| Species | 63.857 | 2 | **< 0.001** | 37.898 | 2 | **< 0.001** |
| Time | 33.494 | 1 | **< 0.001** | 16.759 | 1 | **< 0.001** |
| **Removed terms** |  |  |  |  |  |  |
| Treatment*time*species | 0.174 | 2 | 0.916 | 2.939 | 2 | 0.23 |
| Treatment*time | 1.706 | 1 | 0.191 | 0.089 | 1 | 0.765 |
| Time*species | 3.559 | 2 | 0.168 | 22.359 | 2 | **< 0.001** |
| Treatment*species | 0.276 | 2 | 0.871 | 0.052 | 2 | 0.974 |

Table S5. Effects of species, temperature treatment and incubation time (short or long exposure) and their interactions in latency time and number of tongue flicks in the high altitude populations of the three studied species (results from GLMM).

| High altitude population (pooling together the three species studied) | | | | | | | | |
| --- | --- | --- | --- | --- | --- | --- | --- | --- |
|  | Latency time | | | | Number of tongue flicks | | | |
| **Factors** | **Estimate** | **SE** | **z** | **p** | **Estimate** | **SE** | **z** | **p** |
| Intercept | 2.285 | 0.245 | 9.314 | **< 0.001** | 1.835 | 0.137 | 13.402 | **< 0.001** |
| Treatment hot | 0.598 | 0.346 | 1.726 | 0.084 | -0.596 | 0.222 | -2.689 | **0.007** |
| Species *I. monticola* | -1.368 | 0.435 | -3.143 | **0.002** | 1.084 | 0.191 | 5.689 | **< 0.001** |
| Species *P. muralis* | -0.964 | 0.385 | -2.504 | **0.012** | 0.529 | 1.867 | 2.83 | **< 0.001** |
| Time (Short exposure) | -1.144 | 0.357 | -3.199 | **0.001** | 0.708 | 0.172 | 4.12 | **< 0.001** |
| Treat Hot * Sp. *I. monticola* | 0.808 | 0.583 | 1.386 | 0.165 | -0.044 | 0.297 | -0.147 | 0.883 |
| Treat Hot * Sp. *P. muralis* | 0.646 | 0.519 | 1.246 | 0.213 | 0.653 | 0.282 | 2.313 | **0.021** |
| Treat Hot * Time | -0.339 | 0.507 | -0.669 | 0.503 | 0.424 | 0.27 | 1.568 | 0.116 |
| Species *I. monticola* * Time | 1.37 | 0.605 | 2.263 | 0.236 | -0.411 | 0.245 | -1.677 | 0.093 |
| Species *P. muralis* * Time | 1.904 | 0.542 | 3.511 | **< 0.001** | -0.755 | 0.252 | -2.998 | **0.002** |
| Treat. Hot * Sp. *I. monticola* * Time | -0.729 | 0.819 | -0.891 | 0.373 | 0.344 | 0.368 | 0.934 | 0.35 |
| Treat. Hot * Sp. *P. muralis* * Time | -1.146 | 0.748 | -1.531 | 0.126 | -0.229 | 0.368 | 0.624 | 0.533 |

Table S6. Results from the reduced models based on Likelihood ratio tests. Effect of the species, temperature treatment and incubation time (short or long exposure) and their interactions on the latency time and tongue flicks in the high altitude populations of the three studied species.

| High altitude population (pooling together the thee species studied) | | | | | | |
| --- | --- | --- | --- | --- | --- | --- |
|  | Latency time | | | Number of tongue flicks | | |
| **Reduced model** |  |  |  |  |  |  |
|  | ***X^2^*** | **df** | **p** | ***X^2^*** | **df** | **p** |
| Treatment | 9.295 | 1 | **0.002** | 2.032 | 1 | 0.154 |
| Species | 5.916 | 2 | 0.052 | 76.398 | 2 | **< 0.001** |
| Time | 13.176 | 1 | **< 0.001** | 38.106 | 1 | **< 0.001** |
| **Removed terms** |  |  |  |  |  |  |
| Treatment*time*species | 2.406 | 2 | 0.300 | 2.615 | 2 | 0.270 |
| Treatment*time | 7.421 | 1 | **0.006** | 6.901 | 1 | **0.009** |
| Time*species | 10.49 | 2 | **0.005** | 17.405 | 2 | **< 0.001** |
| Treatment*species | 1.194 | 2 | 0.550 | 8.701 | 2 | **0.013** |
